# Predicting Surface Roughness in Turning Complex-Structured Workpieces Using Vibration-Signal-Based Gaussian Process Regression

**DOI:** 10.3390/s24072117

**Published:** 2024-03-26

**Authors:** Jianyong Chen, Jiayao Lin, Ming Zhang, Qizhe Lin

**Affiliations:** 1 College of Computer Science and Artificial Intelligence, Wenzhou University, Wenzhou 325035, China; 00141027@wzu.edu.cn; 2 Pingyang Institute of Intelligent Manufacturing, Wenzhou University, Wenzhou 325400, China; ljy3326126@163.com; 3 Ebara Great Pumps Co., Ltd., Wenzhou 325200, China; zhangm@ebaragreat.com; 4 College of Mechanical and Electrical Engineering, Wenzhou University, Wenzhou 325035, China

**Keywords:** surface roughness, vibration signal analysis, Gaussian Process Regression, Daubechies Wavelet Packet Transform, complex-structured workpieces

## Abstract

Surface roughness prediction is a pivotal aspect of the manufacturing industry, as it directly influences product quality and process optimization. This study introduces a predictive model for surface roughness in the turning of complex-structured workpieces utilizing Gaussian Process Regression (GPR) informed by vibration signals. The model captures parameters from both the time and frequency domains of the turning tool, encompassing the mean, median, standard deviation (STD), and root mean square (RMS) values. The signal is from the time to frequency domain and it is executed using Welch’s method complemented by time–frequency domain analysis employing three levels of Daubechies Wavelet Packet Transform (WPT). The selected features are then utilized as inputs for the GPR model to forecast surface roughness. Empirical evidence indicates that the GPR model can accurately predict the surface roughness of turned complex-structured workpieces. This predictive strategy has the potential to improve product quality, streamline manufacturing processes, and minimize waste within the industry.

## 1. Introduction

Surface roughness is a critical determinant of product quality, functionality, and reliability in the manufacturing industry. It quantifies the deviation of a workpiece’s actual surface from its ideal form and is influenced by various factors, such as cutting parameters, tool geometry, material, and machining processes. The complexity of manufacturing intricate geometric parts often involves multiple steps, including boring, milling, roughing, and finishing, with surface roughness needing to meet assembly process specifications post-finishing. Achieving an optimal balance of surface roughness is essential, as it can be neither too low nor too high. Consequently, surface roughness is a vital metric for assessing product quality. Traditionally, quality assurance has relied on periodic sampling and manual inspection, which can lead to significant waste when surface roughness fails to meet standards. Thus, the development of a real-time, accurate surface roughness prediction method is imperative.

Previous research has investigated artificial intelligence (AI) approaches for predicting surface roughness in turning processes. Benardos and Vosniakos [1] found AI methods to outperform traditional approaches, while Özel and Karpat [2] and Palanisamy and Shanmugasundaram [3] reported that artificial neural networks (ANNs) surpassed regression models and response surface methodologies. 

Researchers have also refined input variables to enhance prediction accuracy. For example, in 2010, T. Reddy and C. Reddy [4] correlated acoustic emission (AE) with surface roughness, Marani et al. [5] evaluated feed rate and cutting speed in adaptive neuro-fuzzy inference systems (ANFIS), and Lin et al. [6] demonstrated that ANNs could improve prediction accuracy by incorporating vibration signals. Vasanth et al. [7] fused cutting force, tool wear, displacement of tool vibration, and three cutting parameters to predict the roughness in ANN. Moreover, researchers have improved prediction accuracy by enhancing models’ capabilities. Pimenov et al. [8] found that random forests outperformed other models, while Badiger et al. [9] and Kong et al. [10] utilized ANNs and machine learning techniques to optimize cutting parameters and feature extraction, respectively. Su et al. [11] and Lu et al. [12] applied SVM and GPR, respectively, achieving promising results.

Despite these efforts, existing methods, which primarily rely on traditional statistics, SVM, and ANN, depend heavily on comprehensive training data. The complex machining environment, including the use of cutting coolants, often hinders the acquisition of real surface images, necessitating a reliable and accessible method for initial data collection. 

Time domain signal processing methodologies, encompassing statistical functions and advanced techniques, have been extensively investigated for tool condition monitoring in turning processes. Siddhpura and Paurobally [13] conducted a review of flank wear prediction methods, while Elangovan [14] enhanced the selection of statistical features from vibration signals using principal component analysis (PCA), achieving a classification accuracy of 91.2% for flank wear. Aghdam et al. [15] employed the autoregressive moving average (ARMA) model to identify wear-sensitive features based on the dynamics of the tool-holder system, addressing the challenges of identifying random tool wear features in time domain analysis, as noted by Siddhpura and Paurobally [13], Nath [16], and Kuntoğlu et al. [17].

Frequency domain analysis methods, such as FFT and PSD, have been explored by Fang et al. [18] and Plaza et al. [19]. Bhuiyan and Choudhury [20] utilized the root mean square (RMS) of vibration signals and frequency analysis to assess machine tools under various cutting conditions. Tangjitsitcharoen and Lohasiriwat [21] differentiated broken chip signals from tool wear using the PSD of decomposed cutting force.

In the field of machinery fault diagnosis and tool condition monitoring (TCM), the Daubechies Wavelet Packet Transform (WPT) has been recognized for its efficacy in processing vibration signals. The WPT’s selection is attributed to its ability to provide a balanced time–frequency resolution, which is crucial for capturing the transient characteristics of non-stationary signals, such as those encountered in turning processes. Scheffer and Heyns [22], Wang et al. [23,24], and Segreto et al. [25] applied wavelet analysis to classify tool wear, achieving high success rates. The WPT’s adaptability allows for the extraction of features that are highly relevant to the prediction of tool wear and surface roughness. This is demonstrated by the work of Plaza and López [26,27], who established clear criteria for WPT based on vibration signals, and Benkedjouh et al. [28], who combined continuous wavelet transform (CWT) with blind source separation (BSS) to estimate the remaining useful life (RUL) of milling tools with high accuracy.

Furthermore, the WPT’s energy localization capabilities enable the precise identification of frequency components and their temporal occurrences, which is beneficial for fault detection and diagnosis (Gangadhar et al. [29]). The robustness of the WPT against noise, a common challenge in practical applications, is highlighted by Du et al. [30], who combined WPT and WT features to predict tool state with high accuracy using a neural network. The WPT’s utility in feature extraction for machine learning models is exemplified by Kong et al. [31], who presented a predictive model for tool wear that integrated Radial-Basis-Function-based principal component analysis (PCA) and relevance vector machine, with features extracted from time domain statistical functions and Daubechies WPT energy using Shannon entropy. This model significantly reduced the root mean square error, showcasing the WPT’s potential for enhancing the performance of predictive models. 

In comparison to wavelet analysis, the Empirical Mode Decomposition (EMD) has seen less use in recent TCM studies. However, its combination with WT and other methods has shown promise in detecting tool wear (Babouri et al. [32], Li et al. [33], Liu et al. [34]). This suggests that while the WPT is a preferred method, alternative techniques like EMD can still contribute to the field. 

In summary, the Daubechies Wavelet Packet Transform’s selection for processing original vibration signals is justified by its adaptability, balanced time–frequency resolution, energy localization, noise robustness, and effectiveness in feature extraction for machine learning models. These attributes make the WPT a valuable tool in the signal processing toolkit for machinery health monitoring and predictive maintenance.

Recent advancements, including the application of AI for predicting surface roughness in additively manufactured components, as demonstrated by Temesgen Batu et al. [35], the utilization of causality-driven feature selection to enhance deep-learning-based models in milling machines by Hyeon-Uk Lee et al. [36], and the investigation of novel parameters in grinding processes by Mohammadjafar Hadad et al. [37], collectively suggest that innovative methodologies can markedly improve predictive accuracy. The enhanced prediction of surface roughness in titanium alloy during abrasive belt grinding, achieved through an advanced Radial Basis Function (RBF) neural network by Kun Shan et al. [38], and the high precision attained by integrating hybrid features with an Improved Sparrow Search Algorithm-Deep Belief Network (ISSA-DBN) for milling die steel P20, as reported by Miaoxian Guo et al. [39], further highlight the efficacy of these cutting-edge approaches.

This paper proposes using Gaussian Process Regression (GPR) based on vibration signals to accurately predict surface roughness in turning complex-structured workpieces. GPR offers several advantages: it provides uncertainty estimates, it is non-parametric and flexible, and it has demonstrated higher accuracy and robustness compared to other methods. In turning processes, vibration signals, which are less susceptible to external noise and easier to install, are analyzed using GPR. This approach leverages the strengths of vibration signals to improve the prediction of surface roughness.

In turning processes, there are various types of signals that can be utilized as inputs for GPR when predicting surface roughness. These signals include commonly used ones, such as acoustic emission (AE) and cutting force. However, these signals have limitations that can affect their accuracy and applicability in different scenarios. On the other hand, vibration signals possess several advantages. Firstly, vibration sensors can be easily installed without the need to damage the original structure of the machine tool. Secondly, vibration signals are less susceptible to external noise and disturbances, resulting in more reliable and accurate predictions of surface roughness. Therefore, in this paper, GPR is employed to analyze vibration signals from turning complex-structured workpieces. 

This paper elucidates the significance of surface roughness in manufacturing and addresses the complexities of its prediction. It presents a methodology encompassing feature extraction, correlation, and the utilization of Gaussian Process Regression (GPR) for predictive modeling (Section 2). The experimental framework, data acquisition, and vibration signature analysis are thoroughly described (Section 3). This paper delves into signal processing methodologies, culminating in GPR-based predictions (Section 4). The Discussion (Section 5) evaluates cross-validation methodologies and interprets the study’s outcomes, while the Conclusion (Section 6) encapsulates the research’s essence and suggests potential research trajectories.

## 2. Methodology

Our methodology is meticulously designed to predict surface roughness in the turning of complex-structured workpieces using Gaussian Process Regression (GPR) informed by vibration signals. The process encompasses three primary components: signal acquisition, feature extraction, and surface roughness prediction. We capture vibration signals from the turning tool in three orthogonal directions throughout the machining process, which are then preprocessed to mitigate noise from extraneous sources. The feature extraction module extracts parameters from these signals, covering both time and frequency domains. Time domain features are derived using statistical measures, such as the mean, maximum, median, standard deviation (STD), and root mean square (RMS). Frequency domain analysis is facilitated by Welch’s method, which transforms the signals from the time domain to the frequency domain, and it is further enhanced by a three-level Daubechies Wavelet Packet Transform (WPT). These features are subsequently utilized to construct the surface roughness prediction model. The GPR model, known for its ability to model nonlinear relationships between inputs and outputs, is trained with a subset of relevant features selected based on their correlation with surface roughness. The model’s performance is optimized using an iterative conjugate gradient method for parameter determination, ensuring a robust prediction model that is both accurate and efficient.

### 2.1. Feature Extraction and Correlation

Feature extraction from vibration signals is a pivotal step in preprocessing aimed at isolating pertinent information that enhances signal quality and processing efficiency. This process encompasses various methodologies, such as statistical characteristics, frequency domain features, time–frequency features, and nonlinear characteristics.

Statistical characteristics involve calculating the mean, variance, standard deviation, and peak values of the vibration signal, which reflect the average vibration level and tool fluctuation. Frequency domain features are derived through Fourier or wavelet transforms, capturing attributes like power spectral density, frequency components, and dominant frequencies, which are essential for analyzing the frequency composition and distribution of the tool’s vibrations.

Welch’s method, a prevalent frequency domain feature extraction technique, employs Fourier-transform-based signal analysis. It segments the raw vibration data, X(n), into L overlapping subsegments, each containing M data points, resulting in a total of N = LM data points. The jth segment is represented as:

(1) $${\;X}_{j}\left(n\right)=x\left(n+jM-M\right),\;0\leq n\leq M,\;1\leq j\leq L$$

Then, a window function w(n) is added to each data segment, and the power spectrum of each segment is calculated. The power spectrum of the jth segment is

(2) $$I_{j}\left(w\right)=\frac{1}{U}{\left|\sum_{n=0}^{M-1}x_{j}\left(n\right)w\left(n\right)e^{-jen}\right|}^{2}$$

where U is the normalization factor:

(3) $$U=\frac{1}{M}\sum_{n=0}^{M-1}w^{2}(n)\;$$

Assuming that the power spectra of each segment are approximately uncorrelated, the power spectral estimate is given by:

(4) $$P_{xx}\left(e^{jw}\right)=\frac{1}{L}\sum_{j=1}^{L}I_{j}(w)$$

The Welch feature extraction method is employed to obtain frequency components and power spectral density, which are instrumental in analyzing the signal’s frequency composition and distribution. This method enhances the accuracy of spectral estimation but may introduce information loss and estimation errors during signal segmentation and windowing. Consequently, careful parameter selection and optimization are essential, and they are tailored to the specific application and data characteristics.

**Figure 1:**
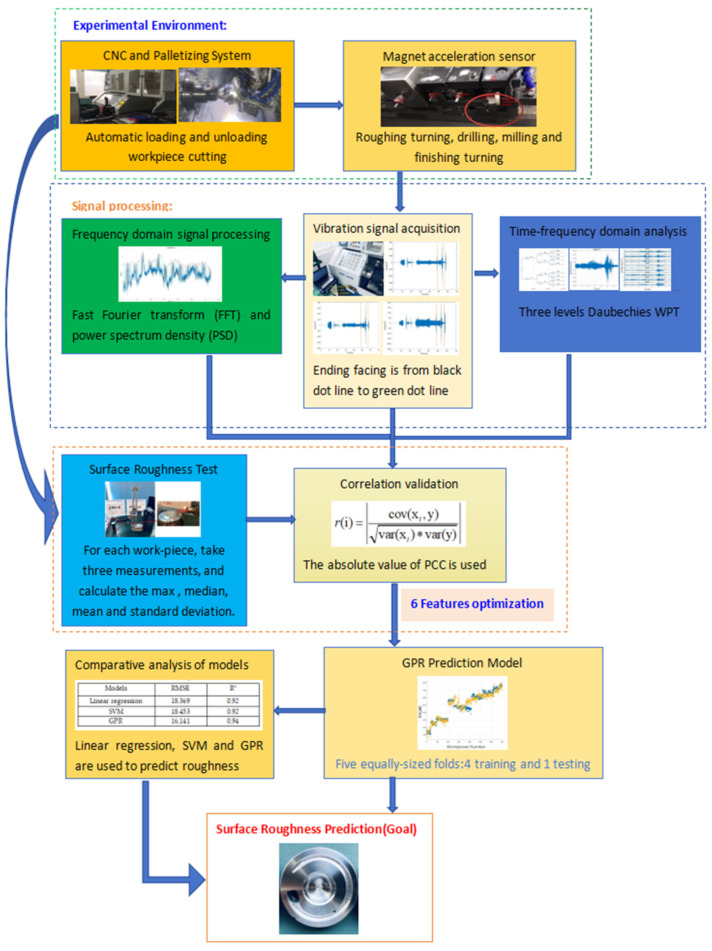
Surface roughness prediction and signal processing workflow.

Time–frequency domain analysis, including short-time Fourier transform (STFT), wavelet transform (WT), Hilbert–Huang transform (HHT), and empirical mode decomposition (EMD), is extensively applied to nonstationary signals for machinery fault diagnosis. In the context of turning process tool condition monitoring (TCM), WT analysis is particularly prevalent due to its significant reduction of processing time and precise identification of specific frequency contributions (Figure 1).

To assess the correlation between the statistical properties of time and frequency domain acceleration signals and roughness data, the Pearson correlation coefficient (PCC) is utilized. The PCC ranges from −1 to 1, with a value of 0 indicating no correlation. Negative values denote an inverse relationship, while positive values indicate a direct correlation. In this study, the absolute value of PCC is prioritized, as higher absolute values suggest stronger correlation features. The mathematical equation for PCC is as follows:

(5) $$PCC\left(i\right)=\left|\frac{cov(X_{i},Ra)}{\sqrt{var\left(X\right)\times var(Ra)}}\right|$$

Here, $X_{i}$ is the ith variable, Ra is the roughness, var represents variance, and cov represents covariance. The value of $PCC\left(i\right)$ represents the correlation between X and Ra.

### 2.2. Gaussian Process Regression (GPR) Model

To predict surface roughness, a subset of relevant features is selected based on actual requirements and the nature of the problem. These features are then incorporated into a prediction model for tool wear degree utilizing machine learning algorithms, such as Support Vector Machine (SVM), decision trees, and neural networks for modeling and prediction.

Gaussian Process Regression (GPR) is a machine learning algorithm adept at modeling nonlinear relationships between inputs and outputs. It uses the chosen features from signal acquisition and feature extraction modules to predict surface roughness. The Gaussian process, represented by Equation (6), is characterized by feature vectors that include certain parameters, such as the time domain maximum, average, median, and root mean square values.

(6) $$f\sim GP[m\left(X\right),\;k(X,X^{\prime })]$$

The mean function, as described by Equation (7), represents the expected value of the function.

(7) $$m\left(X\right)=E(f\left(X\right))$$

The covariance function, detailed in Equation (8), is a critical component of the GPR model.

(8) $$k(X,X^{\prime })=E[(f\left(X\right)-m\left(X^{\prime }\right))(f\left(X^{\prime }\right)-m\left(X^{\prime }\right))]$$

The kernel function of GPR, which combines a mean function and a covariance function, typically sets the mean function to zero. The rational quadratic kernel function, as shown in Equation (9), is a common choice.

(9) $$k(X,X^{\prime })=\sigma^{2}{\left(1+\frac{{\left(X-X^{\prime }\right)}^{2}}{2\alpha s^{2}}\right)}^{-\alpha }$$

This function’s parameters, including the scale factors σ, s, and the proportional mixed factor α, are optimized for this study. The rational quadratic kernel function can be interpreted as a sum of square exponential kernel functions with varying reduction lengths, and it reduces to a square exponential kernel under certain conditions. Its infinite differentiability ensures a smooth function, which is crucial for the GPR model. The optimal parameter solution is determined using an iterative conjugate gradient method.

In essence, a robust prediction model requires accurate signal collection, followed by the optimization and adjustment of feature extraction methods and parameters based on specific vibration signals and tool wear conditions. Additionally, the efficacy of feature extraction and model construction necessitates experimental validation.

## 3. Field Tests and Data Acquisition

### 3.1. Field Experiment Setup

**Figure 2:**
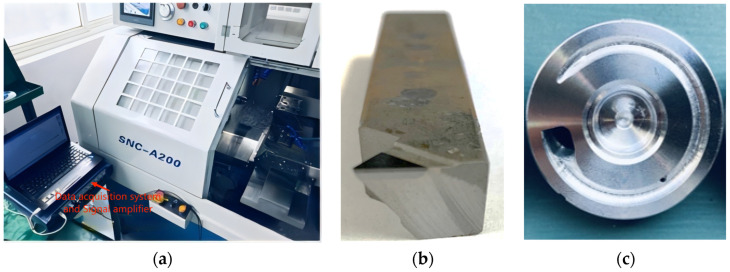
(**a**) The CNC machine; (**b**) the finishing turning tool; (**c**) the workpiece.

**Figure 3:**
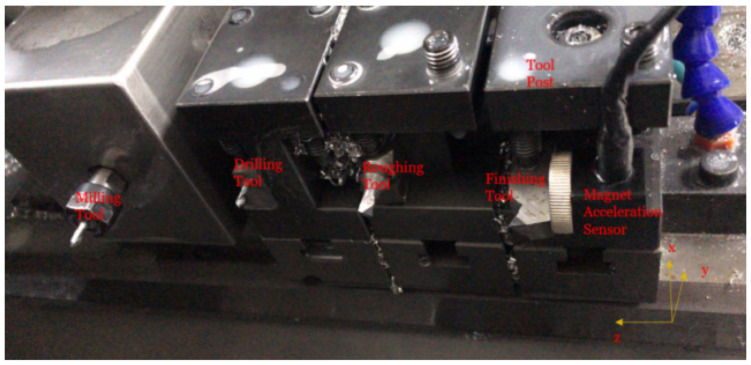
Four machine tools with a magnet acceleration sensor.

The field experiments were conducted using a CNC machine, model SNC-A200, from Shanghai SYMA Machine Tool Technology Corporation, as depicted in Figure 2a. Four types of tools were employed for the machining process: roughing turning, finishing turning, milling, and drilling. The focus of this study is on finishing turning, as it directly impacts surface roughness. The specific tool used is shown in Figure 2b, and a sample of the finished workpiece with a complex structure is presented in Figure 2c. Uniform cutting conditions were maintained for all workpieces, as detailed in Table 1. Figure 3 displays the magnetically attached acceleration sensor on the finishing turning tool.

To ensure high-quality signal preprocessing, several measures were implemented. Prior to collecting vibration signals, the machine tool was meticulously calibrated to eliminate external noise and other factors that could compromise the accuracy of the signals. This included the precise placement of the sensor on the machine tool, positioning it as close to the tool’s head as possible, and adjusting the sensor’s orientation to align with the actual working conditions. Furthermore, the sensor and tool were securely affixed using strong glue and a powerful magnet. After careful adjustment, the sensor position, as shown in Figure 3, was finalized.

During the experiment, vibration signals from 50 workpieces were recorded, along with their surface roughness measurements, over a period of cutting 1000 workpieces. An increase in the number of workpieces processed corresponded to an increase in surface roughness.

### 3.2. Description of Data

The correlation between the workpiece’s surface roughness and the vibration signals during turning is presented below.

#### 3.2.1. Roughness

**Figure 4:**
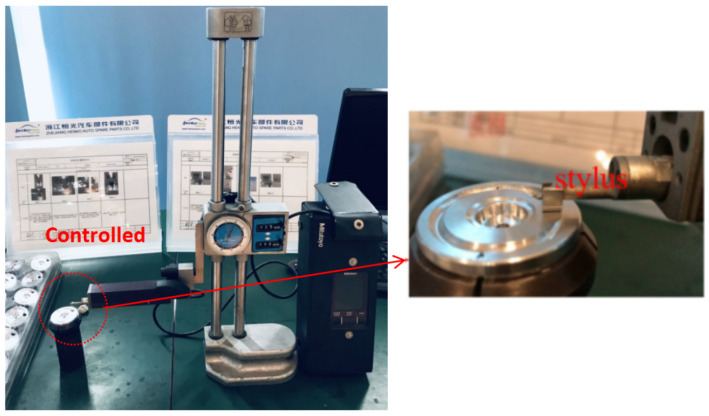
Surfagauge measures roughness.

The workpiece, constructed from ADC-6 aluminum alloy die casting, underwent surface roughness measurements at three distinct surface locations using the Surftest SJ-210 Surfagauge, as shown in Figure 4. The Surfagauge’s stylus was employed to meticulously assess the workpiece’s surface, with the Ra value representing the arithmetic mean of the roughness profile. The maximum permissible Ra value for this workpiece is established at 700 μm.

The roughness values are described as:

(10) $$r_{j}=[r_{j1},r_{j2},r_{j3}]$$

The measured values were taken at three distinct positions on each workpiece. Similarly, the maximum, median, mean, and standard deviation (STD) roughness values were calculated from three separate measurements for each workpiece.

During the SYMA field test, No. 1 Tool was utilized for 62 days, during which it processed a total of 40,060 workpieces. Some of the workpieces exhibited roughness levels exceeding the specified tolerance of 600 µm. Owing to nightly downtime, the daily production ranged from 500 to 1600 workpieces, with acceleration data being collected from approximately 30 workpieces each day. No. 2 Tool was under same cutting parameters as No. 1 Tool. However, No. 2 Tool only cut 29,196 workpieces that reached the desired tolerance.

#### 3.2.2. Vibration Data

The vibration sensor utilized is the CA-YD-3193 from Sinocera Piezotronics Inc. (Yangzhou, China), featuring a sample frequency of 25,600 Hz. The cutting process for a single workpiece encompasses loading (approximate time), rough turning, drilling, milling, finishing turning, and unloading, totaling an approximate cutting time of 50 s. Further details are provided in Table 2.

### 3.3. Vibration Signature

#### 3.3.1. Time Domain Vibration for Cutting One Workpiece

**Figure 5:**
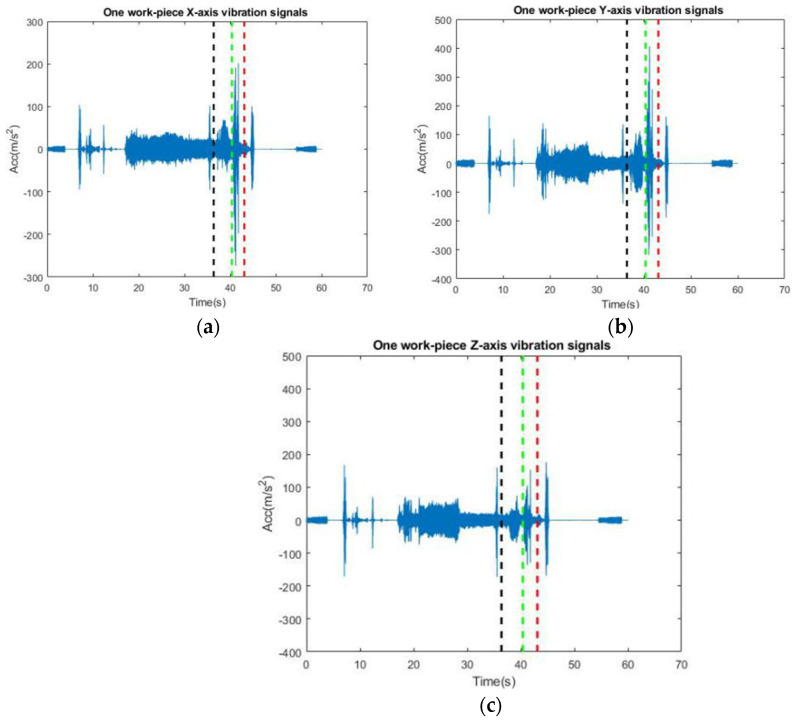
Measured three-axis acceleration of the tool when cutting one workpiece. (**a**) *X*-axis vibration signal, (**b**) *Y*-axis vibration signal, (**c**) *Z*-axis vibration signal.

**Figure 6:**
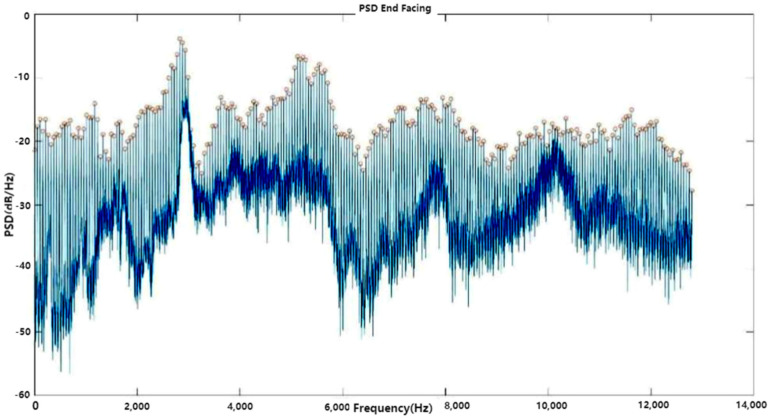
Frequency analysis of finishing turning signals.

Vibration measurements were conducted using a triaxial accelerometer, with the measurement directions specified in Figure 3. Table 2 outlines the procedures in chronological order. Figure 5 presents the vibration signals along the *X*, *Y*, and *Z* axes, as indicated in a–c, respectively. The *Y*-axis displays the highest acceleration values, followed by the *X* and *Z* axes. For this study, only the finishing turning process, which is most relevant to surface roughness, was selected. This is represented by the vibration signal between the two orange, dashed lines in Figure 6.

#### 3.3.2. Frequency Analysis of Finishing Turning Signals

Employing Welch’s method, the power spectrum density of the finishing turning process was obtained and is depicted in Figure 6. The red circles in the figure denote the harmonic peaks of the signal. The fundamental frequency of the vibration was determined to be 53.3 Hz, corresponding to the CNC spindle speed of 3200 RPM.

## 4. Signal Processing and Features Optimization

### 4.1. Time Domain Analysis

The commonly utilized statistical functions in time domain analysis encompass a range of well-established metrics, such as the mean, root mean square (RMS), standard deviation (STD), skewness, kurtosis, and crest factor. These functions are instrumental in characterizing the behavior of signals and identifying trends or anomalies within the data.

The time domain analysis involved the examination of signals from both ending facing and cylindrical turning processes while focusing on certain parameters, such as the mean, RMS, STD, skewness, kurtosis, and crest factor. The statistical properties were categorized into three distinct groups based on the production timeline. The first group spanned from the 2nd to the 17th day, with approximately 5000 workpieces produced; the second group from the 23rd to the 50th day; and the third group on the 62nd day. Over the 62-day sample period, the analysis revealed fluctuations in amplitude with minimal changes in skewness. The mean value, RMS, and STD decreased in the first group, while the second group showed an increase, and the crest factor and kurtosis exhibited a contrasting trend. The RMS and STD properties were notably similar.

**Figure 7:**
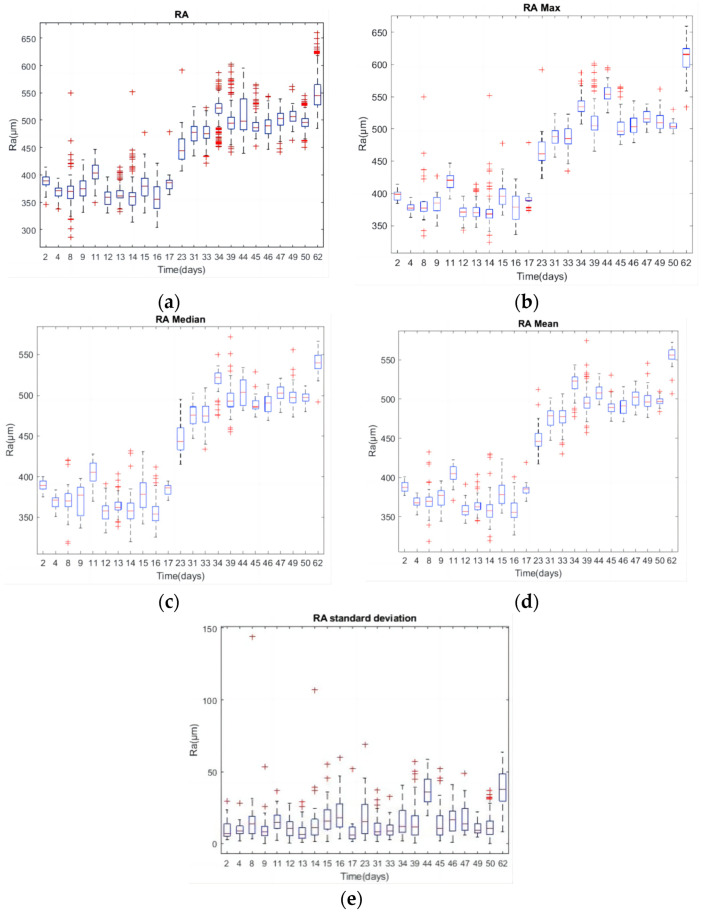
(**a**) Three times of Ra; (**b**) max of Ra; (**c**) median of Ra; (**d**) mean of Ra; (**e**) STD of Ra.

Figure 7a presents roughness measurements from three different positions of the 30 workpieces for each day, with Figure 7b,e depicting the max, median, mean, and STD of these measurements. The roughness values fluctuate, with some abnormal peaks, but generally trend upwards over time. The roughness can be divided into three groups. The first is from the 2nd to the 17th day, with values primarily between 350 µm and 450 µm; the second is from the 23rd to the 50th day, with values around 450 µm to 550 µm; and the third is on the 62nd day, with some workpieces exceeding the desired tolerance of 600 µm.

**Figure 8:**
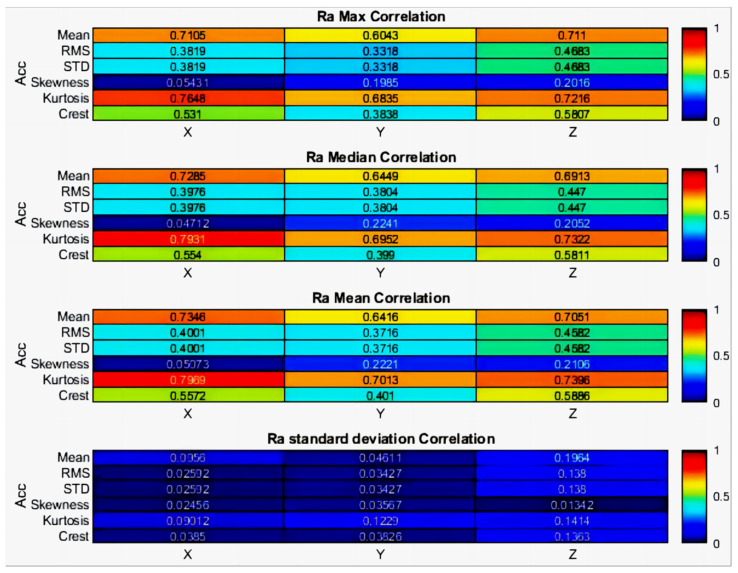
Correlations between statistical properties of time domain and roughness.

The STD, as shown in Figure 7b, occasionally highlighted discrepancies among the roughness measurements. Figure 8’s heatmaps illustrate the PCCs between the time domain’s statistical properties and roughness metrics. The mean value and kurtosis of the time domain in the *X*-axis (tangential direction) and *Z*-axis (feed direction) showed stronger correlations with the max, median, and mean of roughness, with PCC absolute values ranging from 0.70 to 0.79. The kurtosis of the time domain in the *X*-axis demonstrated the highest correlation with each statistical roughness, with a maximum PCC of 0.7969 for the mean roughness value.

In summary, the mean value of the time domain signals in the *X*-axis and *Z*-axis demonstrated consistent performance across both tools, suggesting their potential as relevant features for predicting roughness.

### 4.2. Frequency Domain Analysis

The Fast Fourier Transform (FFT) is a highly regarded and widely applied technique for processing signals in the frequency domain by harnessing the Fourier Transform (FT) to seamlessly transition signals from the time domain to the frequency domain. The FFT algorithm achieves this by performing a series of multiplications and additions, which markedly decreases the computational burden compared to the traditional DFT. This optimization significantly enhances the efficiency of the DFT computation by minimizing the number of complex operations required.

While the FT is fundamental to signal analysis, it faces a limitation due to its reliance on sinusoidal basis functions. This dependency can impede the precise determination of FT coefficients for non-stationary signals, which lack a constant frequency throughout their duration.

Power spectral density (PSD) quantifies the distribution of signal power across various frequency bands. Welch’s method enhances PSD analysis by introducing window functions, which effectively mitigate the noise associated with finite data sets, thus providing a more precise depiction of the signal’s power distribution across frequencies.

**Figure 9:**
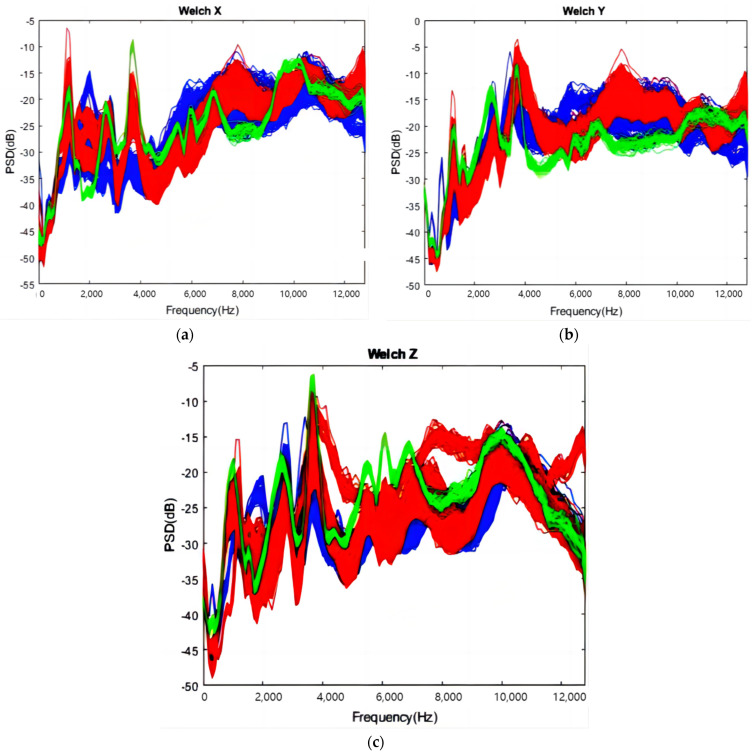
Welch results: (**a**) *X*-axial; (**b**) *Y*-axial; (**c**) *Z*-axial.

Figure 9 demonstrates the application of Welch’s method to analyze the frequency domain of acceleration signals in three orthogonal directions, with a sample rate of 25,600 Hz. The data are divided into 256 overlapping segments, each processed with a Hamming window. The PSD is depicted for the three axes—*X*, *Y*, and *Z*—represented in blue, red, and green, respectively. Typically, PSD increases with frequency, with distinct peaks observed around 3700 Hz for the *X*-axis (Figure 9a) and *Z*-axis (Figure 9c) in Groups 1 and 3. The *Y*-axis (Figure 9b) for Group 2 exhibits the highest PSD at approximately 3700 Hz.

**Figure 10:**
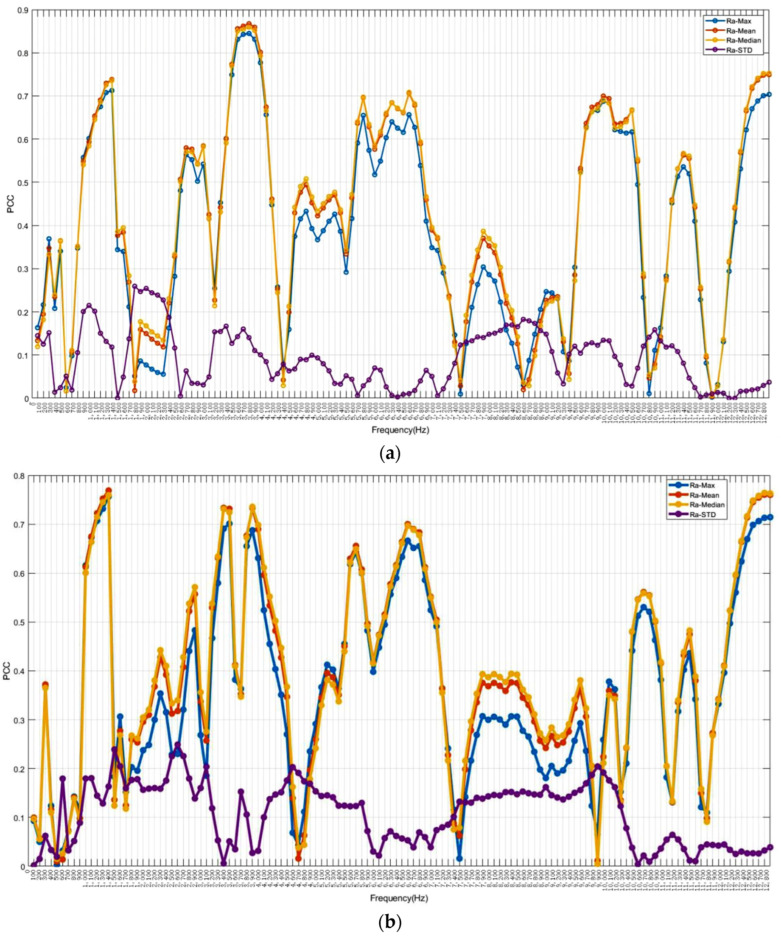

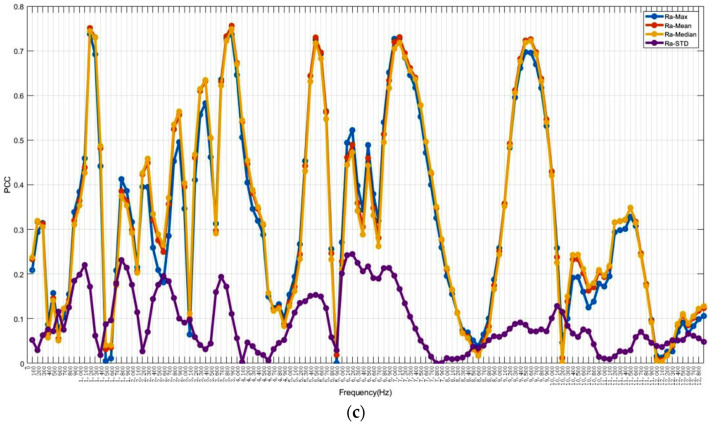
Correlations between roughness and frequency domain: (**a**) *X*-axial; (**b**) *Y*-axial; (**c**) *Z*-axial.

Figure 10 presents the Pearson correlation coefficients (PCCs) between the PSD of every 100 frequencies and the statistical roughness, which aids in the selection of relevant frequencies. The blue, red, yellow, and purple curves represent the max, mean, median, and STD of roughness, respectively. The largest PCCs in the *X*-axis are approximately 0.8446, 0.8674, and 0.8595 for the maximum values, mean values, and median values of roughness, respectively, at 3700 Hz (Figure 10a). Although these PCCs are not the highest in the *Y*-axis (Figure 10b) or *Z*-axis (Figure 10c), they still exhibit strong correlations of around 0.67 and 0.72, respectively. The PCCs for mean values, median, and max of roughness are similar, with the median roughness PCC slightly higher. The highest PCCs are around 0.7689 at 1300 Hz for the *Y*-axis and around 0.7562 at 3800 Hz for the *Z*-axis. Other significant PCCs are observed around 1200 Hz, 6500 Hz, and 12,600 Hz for the *X* and *Y* axes. The STD of roughness is less related to the frequency domain.

**Figure 11:**
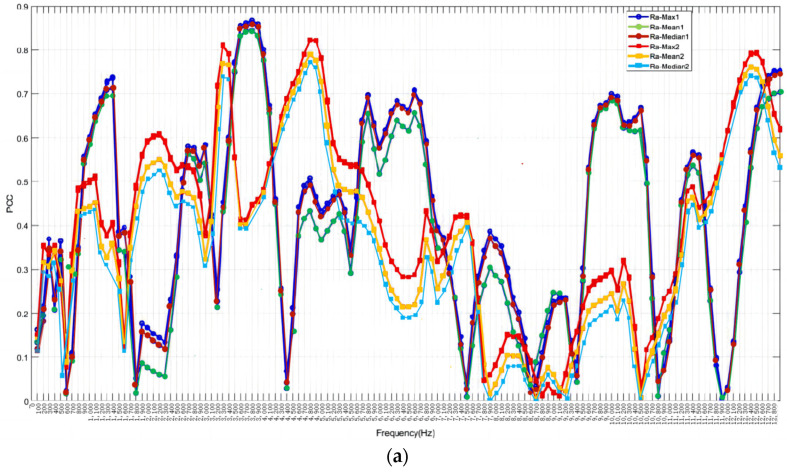

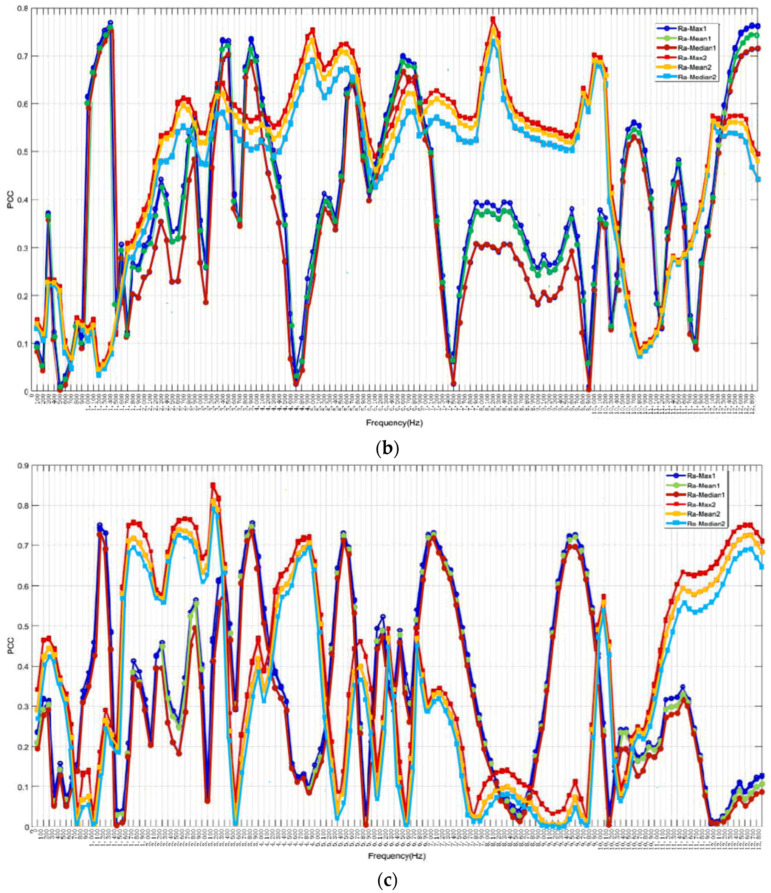
Comparison PCCs of frequency domain and roughness for No. 1 Tool with No. 2 Tool: (**a**) *X*-axial; (**b**) *Y*-axial; (**c**) *Z*-axial.

Figure 11 also shows the comparison of PCCs between the frequency domain and roughness for Tool No. 1 and No. 2. The STD of roughness, which is less related to the frequency domain, is disregarded. The blue and red curves represent Tool No. 1 and No. 2, respectively. For No. 2, the highest PCCs are around 0.8221 at 4700 Hz for the X-axis, 0.7749 at 8100 Hz for the Y-axis, and 0.8511 at 3100 Hz for the Z-axis. The PCCs between the frequency domain and max, mean value, and median of roughness for both tools exhibit similar trends in low frequencies across all three axes. The ranges from 3000 Hz to 4000 Hz generally exhibit steady high PCCs for both tools, especially in the *X*-axis. The PCCs between the frequency domain and roughness are consistently higher than 0.7 around 12,600 Hz for both tools in the *X*-axis.

Table 3 summarizes the comparison of maximum PCCs for time and frequency domains for both tools. The frequency domain PCCs are superior to those of the time domain for both tools.

In summary, the correlation between the frequency domain and surface roughness outperforms that of the time domain, particularly in the frequency range around 12,600 Hz for the *X*-axis, where the PCC exceeds 0.7, indicating a strong correlation and suggesting these frequencies as promising features for analysis. While the peaks of PCCs in the frequency domain vary between the two tools, the trends in low frequencies are notably similar.

### 4.3. Wavelet Packet Transform

Wavelet analysis employs a set of mother wavelets, such as Haar, Daubechies, symlets, Morlets, and coiflets, to decompose signals into their constituent components. The three principal types of wavelet analysis are the continuous wavelet transform (CWT), discrete wavelet transform (DWT), and wavelet packet transform (WPT). A crucial distinction between the short-time Fourier transform (STFT) and wavelet analysis is that STFT breaks down signals into shorter segments using a window function for DFT computation, while wavelet analysis relies on mother wavelets in place of sinusoidal functions. The WPT extends the capabilities of DWT by providing further decomposition of the approximation and detail signals.

**Figure 12:**
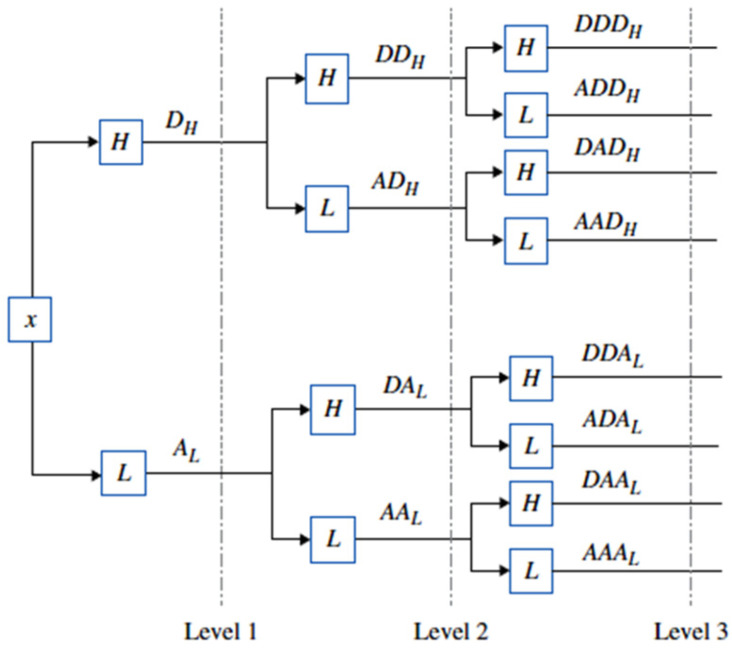
WPT of three levels of decomposition.

Figure 12 presents a three-level WPT decomposition, where the input signal (x) is initially processed by a low-pass filter to obtain the approximation coefficients (AL) and a high-pass filter to extract the detailed coefficients (DH). This process is recursively applied at subsequent levels.

**Figure 13:**
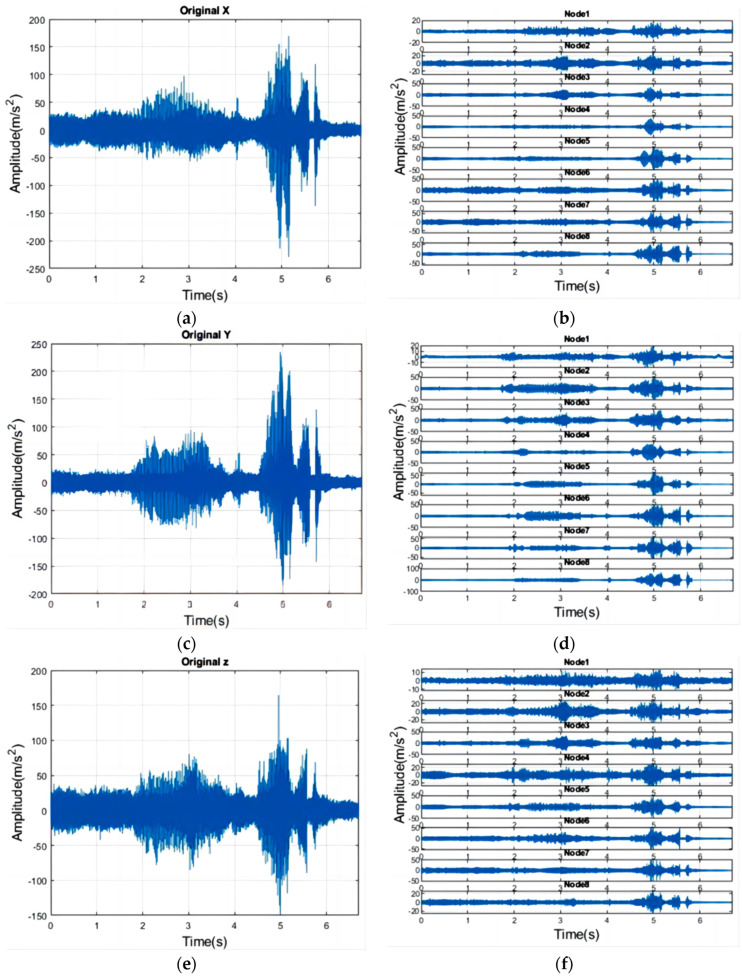
An example of the WPT of three levels of decomposition. (**a**) *X*-axis original vibration signal, (**b**) reconstructions of wavelet coefficient, (**c**) *Y*-axis original vibration signal, (**d**) reconstructions of wavelet coefficient, (**e**) *Z*-axis original vibration signal, (**f**) reconstructions of wavelet coefficient.

Figure 13 provides an example of a workpiece’s signal decomposition using a three-level Daubechies WPT. The original vibration signals for the *X*, *Y*, and *Z* axes are depicted in Figure 13a,c,e, respectively. The corresponding reconstructions of wavelet coefficients are shown in Figure 13b,d,f, where larger nodes represent higher-pass wavelet coefficients, indicating regions of greater frequency content.

**Figure 14:**
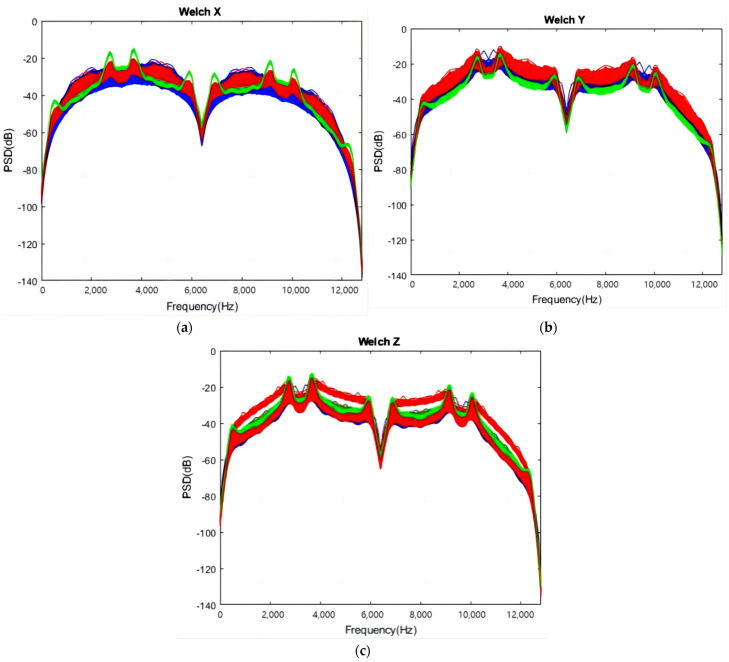
An example of PSDs of wavelet coefficient 3. (**a**) *X*-axis reconstructed coefficient 3, (**b**) *Y*-axis reconstructed coefficient 3, (**c**) *Z*-axis reconstructed coefficient 3.

The PSD results of reconstructed coefficient 3 (frequency range: 3200 Hz to 4800 Hz) are exemplified in Figure 14. Blue, red, and green represent Groups 1, 2, and 3, respectively, consistent with Section 4.1. Notably, there are clear peaks around 3700 Hz in the *X* and *Z* axes for Group 3, similar to Figure 9.

**Figure 15:**
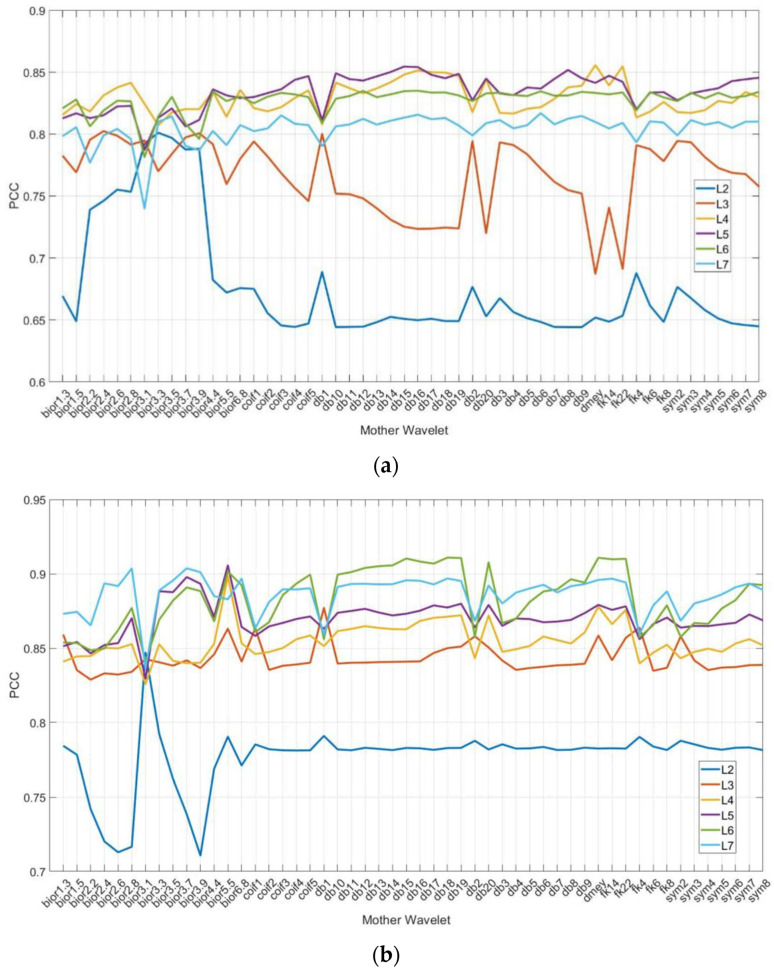
PCCs between energy of each decomposition level (L) and roughness: (**a**) No. 1 Tool; (**b**) No. 2 Tool.

Table 4 lists fifty-two mother wavelet families. Two sets of data are analyzed to determine the optimal performance of PCCs between the energy percentage of these wavelet coefficients and surface roughness, as shown in Figure 15a for Tool No. 1 and Figure 15b for Tool No. 2. Stability-wise, levels L2 and L3 exhibit variability, and their PCCs are lower than other levels for both tools. The best mother wavelet for Tool No. 1 is dmey at level L4 with a PCC of 0.8558 in Figure 15a. For Tool No. 2, the best mother wavelet is db18 at level L6 with a PCC of 0.9109 in Figure 15b.

**Figure 16:**
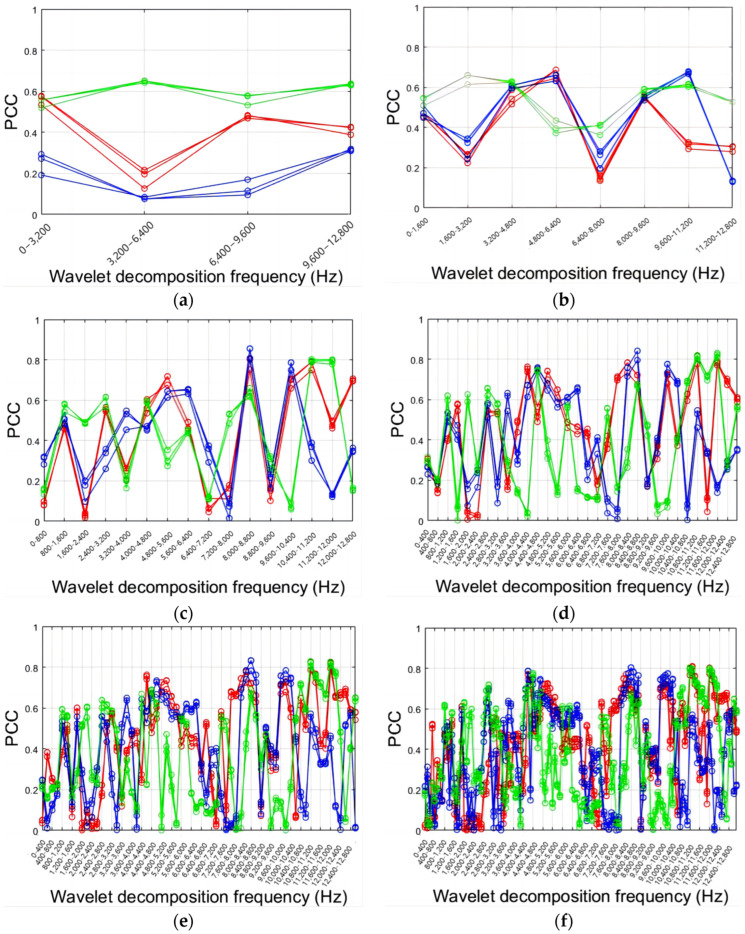
PCCs between energy of each decomposition level (L) and roughness: (**a**) L2; (**b**) L3; (**c**) L4; (**d**) L5; (**e**) L6; (**f**) L7.

Figure 16 explores the effect of decomposition level (L) on the PCCs between the energy percentage of wavelet decomposition and the roughness of Tool No. 1 in three directions. The highest PCC is 0.8558 at 8000 Hz to 8800 Hz in *Y*-axis level L4 in Figure 16d. Low-pass coefficients of scale functions and high-pass coefficients of wavelet functions fail to filter signals correctly at L2 and L3. Changes in energy percentage at high frequencies are more relevant to roughness at L4, L5, L6, and L7. However, increasing decomposition levels does not improve PCCs, as L7’s high-frequency PCC is lower than L5 and L6. There are no significant differences between L5, L6, and L7.

**Figure 17:**
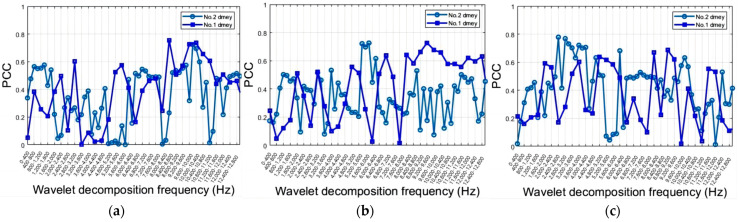
(**a**–**c**) PCCs between energy of dmey decomposition level (L6) and roughness for No. 1 Tool and No. 2 Tool.

Figure 17 compares the PCCs between the energy of dmey decomposition level L6 and roughness for Tool No. 1 and Tool No. 2. The blue line represents Tool No. 1, and the cyan line represents Tool No. 2. PCCs decrease at lower frequencies after WPT in the *X*-axis, while they increase at higher frequencies. The frequency around 10,000 Hz in the *X*-axis is a common feature for both tools, with PCCs around 0.7.

WPT can enhance PCC performance at low and high frequencies, but important features may be filtered during signal decomposition. Increasing decomposition levels does not yield better results, as there are no significant differences between L5, L6, and L7. The best mother wavelet for both tools is dmey.

### 4.4. Results of GPR Predict Roughness

To prevent overfitting due to a large number of features, six optimal features with high correlation to roughness were selected as inputs for the Gaussian Process Regression (GPR) model. The dataset was divided into five equally sized folds, with four folds used for training and one for testing. This cross-validation process was repeated five times while rotating the folds, and the average performance of the model across all folds was calculated to determine the final evaluation model.

**Figure 18:**
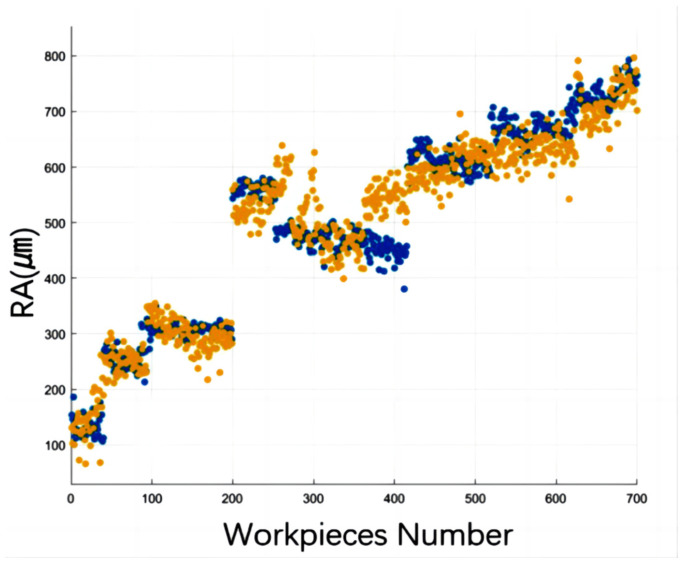
Measured vs. predicted roughness.

Figure 18 shows that the blue dots represent the surface roughness measurements obtained by the Surftest SJ-210 Surfagauge, while the yellow dots indicate the GPR predicted values. The model’s predictions exhibit a strong correlation with the measured results, as demonstrated by an R-squared value of 0.96 and a root mean square error (RMSE) of 35 μm.

## 5. Discussion

The literature review indicates that research on linear regression, Support Vector Machines (SVMs), and Gaussian Process Regression (GPR) models has shown promising results. Consequently, this study undertakes a comparative analysis of these three models as detailed below.

### 5.1. Cross-Validation

Cross-validation is a robust technique for model training and validation, which involves partitioning the training data into two distinct subsets: one for actual training and the other for validation. In the context of k-fold cross-validation, k-1 subsets are designated for training, while the remaining subset serves as the validation set. This process is iterated k times, with each of the k subsets being used as the validation set once. In this thesis, a k-fold cross-validation with k set to five is employed.

The predictive accuracy of the models is assessed using two key metrics: the root mean square error (RMSE), which quantifies the magnitude of the prediction error, and the determination coefficient (R^2^), which measures the degree of correlation between the predicted and actual data.

**Figure 19:**
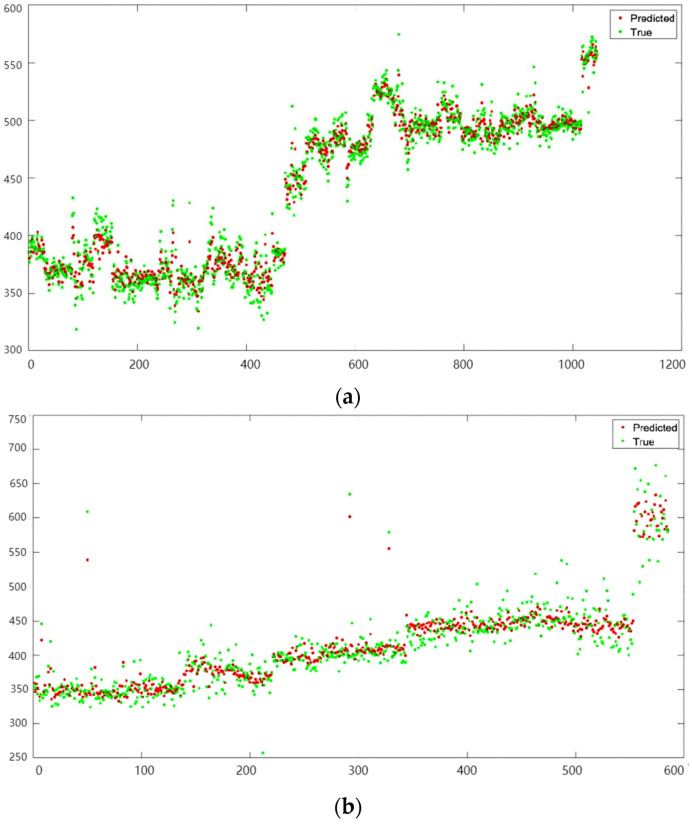
GPR prediction results for (**a**) No. 1 Tool and (**b**) No. 2 Tool.

For predicting surface roughness, linear regression, SVM, and GPR models are applied while utilizing features derived from the preceding sections. Table 5 presents the accuracy metrics for the No. 1 Tool, with GPR emerging as the most accurate model, boasting the lowest RMSE (16.141) and the highest R^2^ (0.94). The GPR model utilizes an exponential kernel function, and the results are visually depicted in Figure 19, where the red points represent the predicted roughness according to GPR, contrasted with the green points representing the measured roughness.

### 5.2. Discussion

In this research, the feature set has been expanded to encompass both time and frequency domains, thereby enhancing the predictive precision of surface roughness estimation.

For the No. 2 Tool, the analysis is conducted using the same input variables and models as for the No. 1 Tool. The comparative results, as presented in Table 5, reveal that the GPR model once again outperforms others with the lowest RMSE (26.682) and the highest R^2^ (0.83). The GPR’s predictive performance is visually demonstrated in Figure 9, where the predicted roughness (red points) is juxtaposed with the measured roughness (green points). The reduced accuracy observed for the No. 2 Tool is attributed to the lower Pearson correlation coefficients (PCCs) of the input variables, indicating a weaker correlation with the output variable.

## 6. Conclusions

This study presents a predictive model for surface roughness in the turning of complex-structured workpieces employing Gaussian Process Regression (GPR) and vibration signals. The model incorporates parameters extracted from the time and frequency domains of vibration signals captured in three orthogonal directions from the cutting tool. These features are then utilized as inputs for GPR to forecast surface roughness. The experimental outcomes confirm that the model delivers precise predictions for surface roughness in complex turning operations. The model’s utility extends to the optimization of cutting parameters, tool geometry, and material selection, enabling the attainment of the desired surface finish within a turning system.

A significant advantage of this approach is its ability to accurately predict surface roughness for intricate workpieces without relying on intricate simulations or laborious physical measurements. This capability can streamline the quality control process in manufacturing, reducing both costs and time, and aid manufacturers in refining their production methods to enhance surface quality and minimize waste.

Furthermore, the model’s predictive capabilities can be harnessed for real-time surface roughness estimation during manufacturing. This real-time feedback allows manufacturers to dynamically adjust machining parameters, ensuring superior surface finish and a lower rate of defective products.

## Figures and Tables

**Table 1:** SYMA CNC cutting parameters.

Cutting Parameters	Data
Depth of cut	0.06 (mm)
Spindle speed	3200 (RPM)
Feed rate	0.06 (mm/rev)
Tool nose radius	0.6 (mm)
Tool overhang length	13 (mm)
Cutting fluid	With coolant

**Table 2:** Approximate time duration of each step of cutting.

1	8~12	Loading
2	15~16	Moving
3	16~20	Roughing
4	21~23	Drilling
5	23~25	Moving
6	25~47	Milling
7	47~48	Moving
8	48~53	Finishing Turning
9	53~58	Moving Back

**Table 3:** Comparison of two tools’ maximum values of PCCs.

Tool No.	Domain	Parameters	*X*	*Y*	*Z*
1	Time	PCC	0.7969	0.7013	0.7396
		Property	Kurtosis	Kurtosis	Kurtosis
	Frequency	PCC	0.8674	0.7689	0.7562
		Frequency (Hz)	3700	1300	3800
2	Time	PCC	0.8267	0.2708	0.7402
		Property	STD/RMS	STD/RMS	STD/RMS
	Frequency	PCC	0.8221	0.7749	0.8511
		Frequency (Hz)	4700	8100	3100

**Table 4:** Fifty-two mother wavelets.

Mother Wavelets Families	Order
Daubechies	db2, db3, db4, db5, db6, db7, db8, db9, db10, db11, db12, db13, db14, db15, db16, db17, db18, db19, db20
Haar	Haar
Discrete Meyer	dmey
Fejer–Korovkin filters	fk4, fk6, fk8, fk14, fk22
Coiflets	coif1, coif2, coif3, coif4, coif5
Symmlets	sym2, sym3, sym4, sym5, sym6, sym7, sym8
Biorthogonal	bior1.3, bior1.5, bior2.2, bior2.4, bior2.6, bior2.8, bior3.1, bior3.3, bior3.5, bior3.7, bior3.9, bior4.4, bior5.5, bior6.8.

**Table 5:** Prediction models’ accuracy.

Models	Tool	RMSE	R^2^
Linear regression	1#	18.369	0.92
	2#	28.19	0.81
SVM	1#	18.453	0.92
	2#	27.779	0.81
GPR	1#	16.141	0.94
	2#	26.682	0.83

## Data Availability

Data are contained within the article.
